# Building on Indigenous Knowledge for Strengthening Food Security and Nutrition: Evidence from a Community-based Intervention in Eastern India

**DOI:** 10.1016/j.cdnut.2026.109434

**Published:** 2026-07-13

**Authors:** Nitya Rao, Arundhita Bhanjdeo, Madhuri Kamtam, Gurpreet Kaur, Tina Banerjee

**Affiliations:** 1 School of Global Development, University of East Anglia, Norwich, UK; 2 Professional Assistance for Development Action (PRADAN), Dumka, India; 3 Independent researcher

**Keywords:** nutrition, knowledge–attitude–practice, community workshops, Santal Parganas, Jharkhand

## Abstract

**Background:**

Indigenous communities in India face high rates of malnutrition despite living in biodiverse regions. Structural drivers, including declining forest cover due to land use changes (mining, urbanization, and infrastructure development), male outmigration, women’s time poverty, and the erosion of intergenerational food knowledge, exacerbate these inequities.

**Objectives:**

The participatory action research project aimed to improve dietary diversity and nutrition among an Indigenous community—the Santals—in 2 development blocks in the states of Bihar and Jharkhand in Eastern India.

**Methods:**

Building on a community-led, culturally grounded food and nutrition intervention, involving the documentation and nutritional analysis of local foods, and development and dissemination of 9 meal templates, data were collected through preinterventional and postinterventional assessment, designed as a knowledge, attitudes, and practices survey, with 684 individual women who were part of self-help groups (SHGs). Alongside this, focus group discussions with SHG members and semistructured interviews with 14 frontline workers and 2 local government (Panchayat) leaders were conducted. Participatory nutrition literacy workshops, recipe-based demonstrations, and training of local women as nutrition champions was undertaken.

**Results:**

Postintervention, nutrition knowledge improved significantly (i.e., awareness of iron for anemia prevention rose from 45%–80%), alongside adoption of diverse, balanced diets. Approximately 65% of the women reported increased meal planning, inclusion of seasonal greens, eggs, local fish, and revived traditional recipes. The intervention strengthened women’s agency, food equity in households, peer-to-peer learning, and intergenerational knowledge exchange. Panchayat engagement and integration with government programs such as “Didi-Bari” (Sister’s garden) supported sustainability. The seasonality of food availability, water scarcity, and women’s time and labor for the collection and preparation of diverse diets remain key constraints.

**Conclusions:**

Community-led, culturally relevant nutrition interventions can enhance dietary diversity, revive Indigenous knowledge, and empower women. Scaling these approaches through policy convergence offers a pathway toward equitable and sustainable food systems.

## Introduction

Food insecurity and malnutrition remain pressing global challenges. Approximately 2.6 billion people are unable to afford a healthy diet, which costs an estimated $4.46 purchasing power parity per person per day, compared with the $3.0 poverty line. Approximately 638 to 720 million people remain undernourished [1,2]. Despite some progress, the world is off-track to achieve the Sustainable Development Goal (SDG) 2 targets on ending hunger and improving nutrition, while simultaneously grappling with the triple planetary crisis of climate change, biodiversity loss, and pollution [3]. Additionally, growing competition for natural resources, new forms of conflict, forced displacement and migration, widening inequalities, declining trust, weakened institutions, and an increasingly multipolar global order are creating a polycrisis with implications for human and planetary health [4].

Indigenous populations are disproportionately affected by these challenges. Paradoxically, although many live in biodiversity-rich regions, they experience marked health and nutrition disparities. For instance, the incidence of type 2 diabetes among American Indian and Alaska Native children and youth aged between 10 and 19 y was 10 times higher than that for their non-Hispanic White counterparts in 2012, as against twice that among adults, and life expectancy 5 to 10 y lower [5,6]. A similar pattern is evident in India. Infant mortality rates among scheduled tribes (STs) in India was 41.6 per 1000 live births in 2019 to 2021 as against 35.2 for nontribes, with Jharkhand, where this study is located, showing a higher gap of 8.7 percentage points [7, see Table 6.1: 126]. Among adults too, one quarter (25.5%) of ST women aged 15 to 49 y had low BMI (in kg/m^2^) and 64% were anemic, both indicators were ∼7 percentage points higher than the aggregate [7, see Tables 6.4 and 6.5, pp 132–133].

Although 90% of “Adivasi” (self-defined term for Indigenous or tribal) populations live in forested areas, >40% lie below the poverty line as compared with 20% of nontribal populations [8]. Malnutrition and communicable diseases, such as malaria and tuberculosis, remain rampant due to poor social and economic access to the required nutrients and health care services. There is simultaneously, however, a growing consumption of cheap, ultraprocessed foods and drinks, which could be contributing to a rising incidence of noncommunicable diseases such as diabetes, hypertension, and mental illness [7,9].

Three rounds of diet and nutrition surveys conducted with the STs by the National Nutrition Monitoring Bureau, in 1985 to 1986, 1997 to 1998, and 2007 to 2008, are instructive. They point to a secular decline in the consumption of roots, tubers, and vegetables [10], more so for men than for women; Over 50% of both adult men and women were below BMI of 18.5 [10]. This decline can partly be explained by the gradual undermining of the diversity of food systems by state policies, including reduced access to forest resources because of land use changes (mining, urbanization, and infrastructure development), delocalization of land governance, and economic frameworks emphasizing market-centric production [6,11]. Dietary diversity is a qualitative measure of food consumption that reflects household access to a variety of foods and is also a proxy for nutrient adequacy of the diet of individuals.

The disruption of “Adivasi” livelihoods has led to an erosion of traditional knowledge systems and, with them, collaboration and benefit-sharing mechanisms that underpin community resilience [12]. This is exacerbated by other factors, including male migration to urban centers in search of employment [13], women’s time poverty resulting from enhanced work burdens in both production and care [14,15], and the lack of recognition of the nutritive values of local foods [16]. Intergenerational transfer of knowledge and practices is also declining, as younger generations, exposed to modern education systems and changing aspirations, are becoming increasingly distanced from their food cultures and resources [focus group discussion (FGD) with adolescent girls during cooking demonstration workshop in Chakai, January 2023). Emerging research highlights the nutritional and cultural significance of Indigenous foods [17,18]. Locally foraged foods, although often stigmatized as wild or inferior by state and professional elites, have been shown to improve dietary diversity among tribal women [6,19,20]. Integrating Indigenous knowledge into food systems therefore holds significant potential to address nutritional inequities, while simultaneously contributing to biodiversity conservation, climate adaptation [21], and giving recognition to Indigenous agency, identity, and wisdom.

This perspective aligns with emerging thinking around food security and nutrition, which moves beyond availability and access to also emphasize the importance of agency and sustainability [22,23]. Drawing on ideas of food sovereignty, which emphasize rights to healthy and culturally appropriate food, produced through ecologically sustainable methods [24], agency implies the capacity of individuals or groups to make decisions about the foods they consume, produce, process, and distribute. It also reflects empowerment or the ability to engage in processes that shape food system policies and governance [22], critical also for sustainability in a context of climate change, resource degradation, and growing social and economic inequalities.

Although food sovereignty among Indigenous peoples is seen to be rooted in cultural knowledge, land stewardship, and self-determination [[23], [25]], evidence suggests that disadvantaged individuals and groups (including women, small farmers, and Indigenous/tribal people) often lack agency with respect to food security, including in regions such as the Santal Parganas [26]. Their access to food is typically mediated through a combination of what they can secure from their own cultivation, the state-sponsored public distribution system, or local markets [26]. Estimates based on wages and recommended dietary intakes suggest that 63% of the rural population in India could not afford a healthy diet in 2015 [27]. This burden is likely to be greater for the STs, as reflected in higher levels of underweight, stunting, and anemia among these children relative to nontribes [7, see Table 6.2, p. 129). Data from a Food Insecurity Experience Scale study conducted by the authors in 2020 across the same and neighboring villages showed that the respondents experience moderate food insecurity—ranging from compromising on the quality and diversity of food to reducing quantities and skipping meals (https://www.fao.org/measuring-hunger/access-to-food/about-the-food-insecurity-experience-scale-(fies)/en). Structural constraints in food access then lead to a denial of food sovereignty and indeed agency and empowerment in relation to food security and nutrition.

Against this backdrop, we initiated an action research project in the Santal Parganas region (Bihar–Jharkhand) of Eastern India to strengthen community nutrition through 2 complementary activities: *1*) documenting traditional Santal recipes and assessing their nutritional values [16] and *2*) developing a community-based recipe book to promote balanced, diverse, and affordable meals [26]. Santhal is a tribal group in India, whose native language is Santhali. Building on this work, conducted in partnership with Professional Assistance for Development Action (PRADAN), a large development nongovernmental organization (NGO) working in the region, the project delivered participatory nutrition workshops to empower communities with practical, culturally grounded dietary solutions.

In this article, we explore the impact of these nutrition workshops on the knowledge, attitudes, and practices (KAP) of women participants, with the objective of generating evidence for community-based nutrition interventions in Indigenous settings. A growing body of literature suggests that nutrition knowledge interventions have positive results on nutritional status [28,29]. Behavior change theories, widely used in public health, provide a range of tools to help identify causal factors and assess behavior along a continuum of change [30]. A study on vegetable consumption in rural Bangladesh, e.g., revealed positive effects on women who had been exposed to individual, role-model stories delivered by a literate community agent [31], whereas Simpson et al. [32] suggest the use of participatory approaches to ensure the sustainability of behavior change. Given our emphasis on ensuring the food sovereignty of the Indigenous communities with whom we were working, providing space for agency and voice, we drew on the theories and experiences of behavior change to design both our intervention and research.

KAP surveys, a widely used tool in nutrition research, help not only assess the communities’ KAP but also identify potential barriers to change, thereby enabling the design of more effective interventions [33]. Guided by the FAO’s KAP framework, we employed a KAP survey covering personal and environmental factors [34]. This helped us develop a comprehensive understanding of the local nutrition context and the personal and social determinants shaping dietary behavior [35].

## Methods

### Intervention design

The intervention was implemented in 2 phases across Chakai (24.56°N, 86.40°E) and Shikaripara (24.24°N, 87.47°E) blocks (subdistrict administrative units) of the Santal Parganas region in Bihar and Jharkhand. In the first phase, a cadre of 10 local youth (6 women and 4 men) were trained as nutrition champions in Chakai. These individuals formed the core of the knowledge exchange process, acting as community leaders and role models to improve dietary practices and promote local food systems [31]. Learnings from phase 1 indicated that nutrition champions could be more effective when connected to structured local institutional networks, so in phase 2, we expanded the model to work through women self-help group (SHG) members. This enabled us to leverage an existing local platform for peer learning, information exchange, and sustained engagement related to the nutrition workshops; 15 local women, who were part of SHGs, were trained in Shikaripara. Together, these 25 nutrition champions were central to the delivery of participatory workshops and sustaining dialogue on food, diet, health, and culture within their communities. These champions also participated in the assessment of changes in community members’ KAPs around food and nutrition using quantitative and qualitative tools.

### Nutrition workshops

Across both phases, a total of 56 participatory workshops were conducted under the banner “Abua Disom Abua Jom” (My land, My food). In phase 1, 24 workshops were conducted with 298 Santal women across 24 villages, whereas phase 2 included 32 workshops with 386 Santal women from 15 villages. The workshops were also attended by children and the elderly in some villages.

The workshops followed our action research protocol, with a structured 2-d format. On day 1, the nutrition champions introduced themselves and the purpose of their visit to the participants and engaged them in a KAP survey. This was followed by general discussions on dietary patterns, nutrition transitions, and barriers to consuming Indigenous foods. Participants were then introduced to the “Santal Recipe Book,” a collection of 9 seasonally categorized recipe templates, combining local dishes with their nutritional values, developed by the project. To address issues of low literacy, this was presented both pictorially and in the local language, Santali [26]. The champions also used printed recipe books, posters, banners, and other locally tailored communication materials for community interactions. To ensure consistency in workshop delivery across villages and nutrition champions, ≥1 coauthor or a member of the local PRADAN implementation team was present during the session. Day 1 ended with the champions and the community participants selecting a menu template for day 2 based on the seasonal availability of food items.

Day 2 was hands-on and participatory, with groups of 10 to 15 women cooking 1 of the selected recipe templates on open cookstoves. The process involved practical learning on healthy cooking practices and culminated in a communal meal with family members and village elders, reinforcing the social and cultural value of shared food. Day 2 ended with participants’ reflections on the taste and their experience with cooking and eating the meal.

In phase 2, additional follow-up visits were conducted with SHGs of women to cover the remaining recipe templates. This adaptation responded to evidence from phase 1, which suggested that a single workshop was insufficient to shift practices. During these follow-up visits, nutrition champions gathered insights on the experiences of and shifts in meal preparation and consumption, including the integration of Indigenous foods in their daily diets. This iterative approach reinforced knowledge retention, improved recall, and helped address barriers such as time constraints in cooking, children’s disinterest, stigma around Indigenous foods, and the easy availability of processed alternatives such as bread and packaged snacks. In 1 case, recipes were also introduced within local “Anganwadis” (childcare centers), where children (aged ≤6 y) consumed dishes prepared by the Anganwadi workers as part of their daily meals.

### Capacity building of nutrition champions

A total of 25 local youth were trained and continuously mentored as nutrition champions across the 2 phases. Training sessions were complemented by refresher modules and standardized tools to strengthen their capacity as community nutrition champions, reduce social desirability bias, and enhance methodological reflexivity. Several were subsequently integrated into PRADAN’s broader health and nutrition programs, becoming part of a regional resource pool supporting participatory research and community development.

### Data collection and participant profile

A KAP survey tool tailored to the local context was developed in line with widely used community nutrition KAP frameworks [[34], [35], [36], [37]]. On day 1 of the workshops, preinterventional surveys, designed in English, were translated and administered in the local language by the trained nutrition champions using a paper-based format. This was conducted face-to-face with the participants, taking ∼20 to 30 min per participant. Follow-up postinterventional surveys were conducted with the same respondents ∼4 mo after the workshops to capture changes over time.

The final sample included 288 participants from Chakai (across 3 Panchayats) and 207 participants from Shikaripara (across 9 Panchayats). The majority were adult married women, seen as responsible for household nutrition. Basic demographic data of participants are presented in Supplemental Table 1, demonstrating overall low educational levels and a primary dependence on agriculture. Details for Chakai and Shikaripara blocks are in Supplemental Table 2.

Alongside the structured surveys, qualitative data were collected by the authors through 5 FGDs involving ∼40 to 50 women in total, as well as through informal reflections documented during the workshops. The FGDs were conducted with SHG members and nutrition champions in villages, demonstrating significant positive changes as well as those facing moderate or limited uptake of the intervention. Informal reflections emerged organically during the nutrition workshops, as participants shared their experiences of cooking and consuming the recipes, barriers to adoption, along with their feedback on taste and food preferences. Further qualitative data were gathered through semistructured interviews with 6 Anganwadi workers, 5 Accredited Social Health Activists, and 3 Poshan Sakhis (community nutrition workers), and key informant interviews with 2 Panchayat leaders. Please see Supplemental Annex 1 for survey and qualitative tools used. Field notes were recorded, when possible, and coded thematically. The following 4 broad thematic domains guided the analysis: *1*) impact on awareness and attitudes; *2*) food and nutrition availability; *3*) access to foods and financial resources; and *4*) perceived impacts on health outcomes.

Ethics approval for the study was obtained from the University of East Anglia’s Development Ethics Committee. Informed consent was obtained from all participants before the surveys and FGDs. In line with our ethics protocol, names of Panchayats, villages, and individuals have been anonymized.

### Data scoring and analysis

Participants’ responses to the surveys were assessed using a standardized KAP scoring system adapted from the literature [38]. The knowledge section had 14 questions, each scored 2 points for a correct or good answer, 1 point for a partially correct or somewhat knowledgeable answer, and 0 points for an incorrect or no knowledge response. Correct and partially correct responses in the knowledge section were determined based on established nutritional science. This produced a possible total score between 0 and 28. The attitudes section included 13 questions, with 2 points given for agree, 1 point for neutral, and 0 points for disagree, creating a total score range of 0 to 26. The practice section contained 10 questions, scored according to how often a behavior was reported: 0 points for never, 1 point for once a week, 2 points for twice a week, and 3 points for >3 times a week. This generated a possible score between 0 and 30. Higher scores indicated greater knowledge, more positive attitudes, or healthier practices. For interpretive reference, scores <50% of the maximum were considered poor, 50% to 75% moderate, and >75% good, consistent with KAP research conventions [38,39].

For each participant, domain scores were calculated by adding the coded responses, converting the categorical answers into continuous values suitable for comparison across time points. Data quality checks were carried out before analysis. Participants with >5 missing responses overall were excluded, whereas those with ≤5 missing answers (51 in Shikaripara and 5 in Chakai, most missing only 1 or 2 items) were retained by imputing values using the mean of their remaining responses in the relevant domain. This is conceptually justified as items within a domain share a common underlying construct. A sensitivity analysis comparing results with and without imputed values confirmed that mean imputation did not materially change the domain score means or overall findings (see Supplemental Table 3 for full comparison of means before and after imputation). Mean scores and SDs were calculated before and after the intervention at both the Panchayat and block levels. The difference between preinterverntion and postintervention means was then used to estimate the change attributable to the nutrition workshops.

### Statistical analysis

Changes in KAP scores were assessed using paired *t* tests, which accounted for the fact that the same participants completed the questionnaire at both preintervention and postintervention points. Separate analyses were performed for each KAP domain within each Panchayat, as well as for block-level aggregated scores. Statistical significance was set at *P* < 0.05. All data coding, cleaning, and analysis were conducted in Stata software, using programmatic commands to ensure reproducibility and consistency.

**FIGURE 1:**
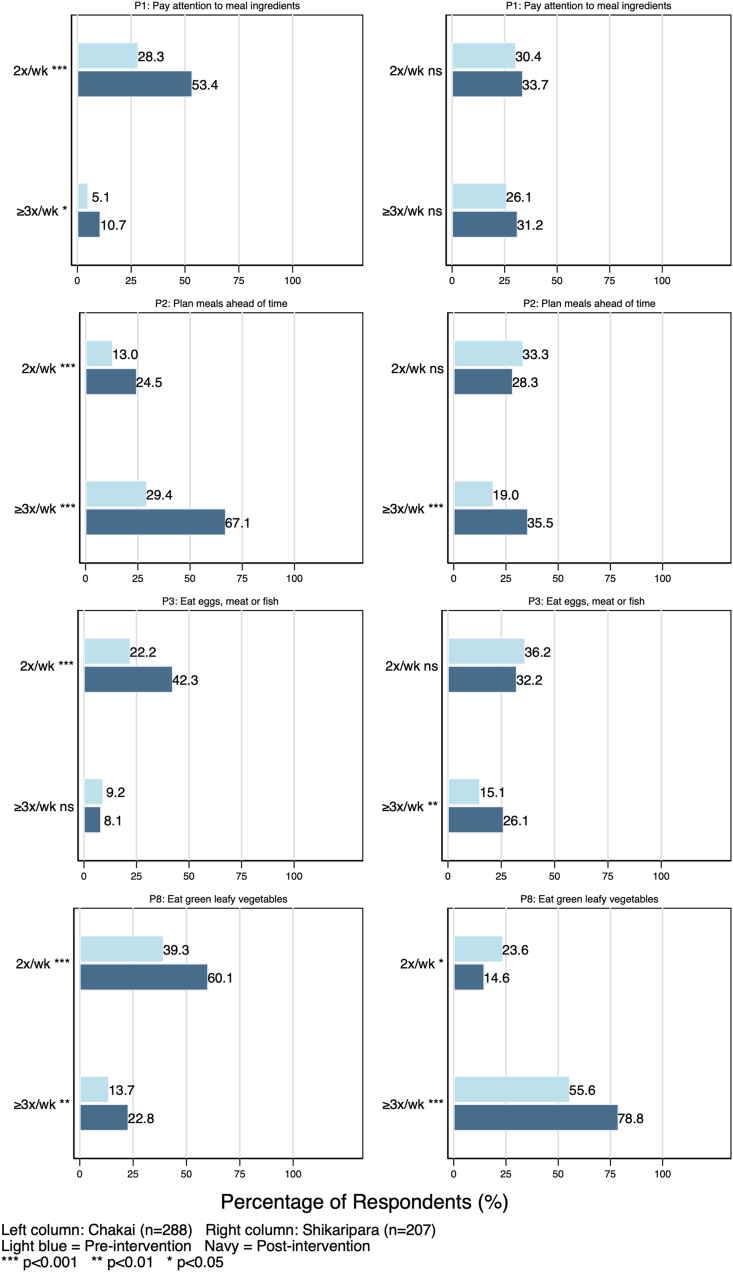
Practive frequency responses for selected indicators: Chakai and Shikaripara.

**FIGURE 2:**
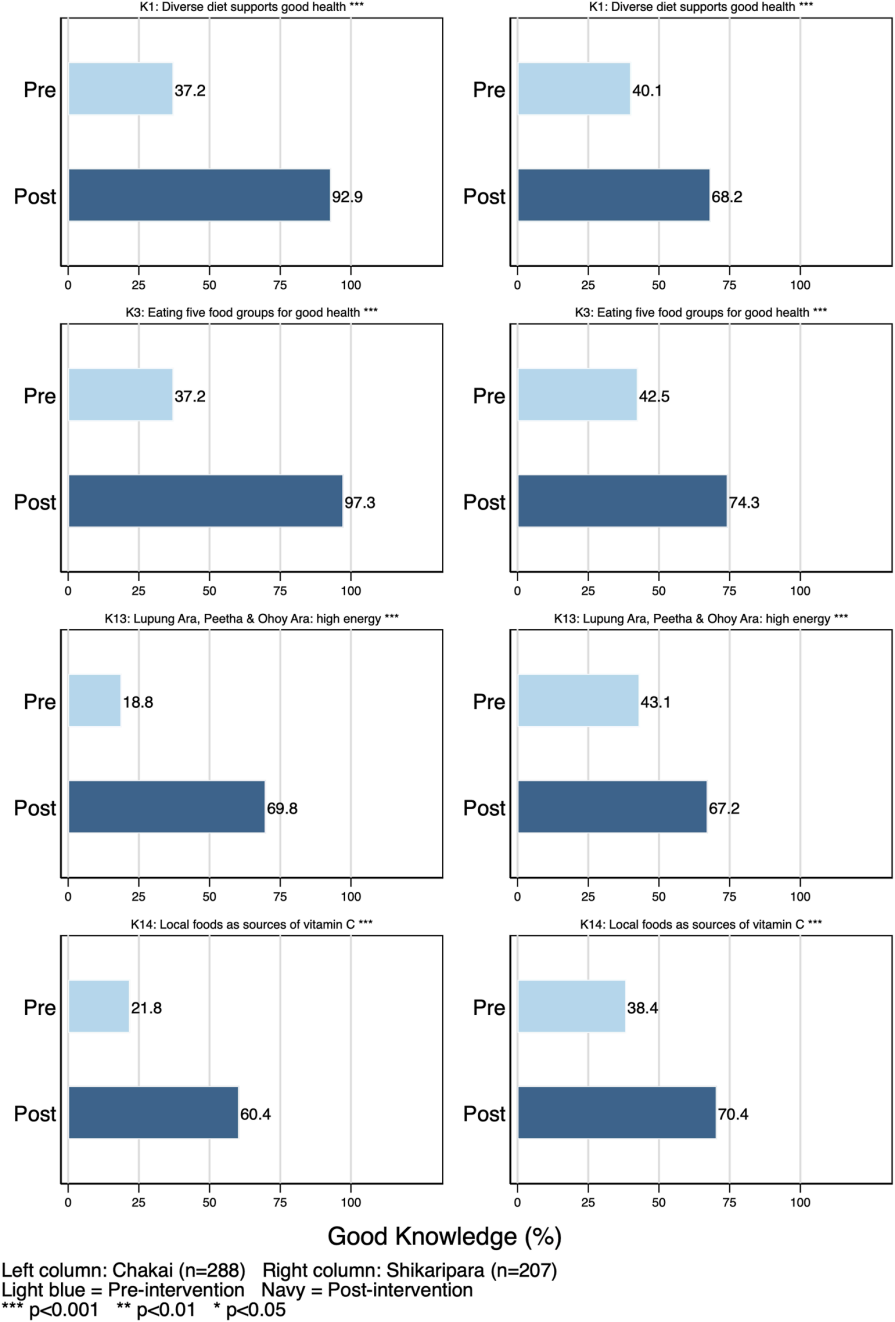
Good knowledge responses for selected indicators: Chakai and Shikaripara.

To examine change at the individual indicator level, 2-proportion *z*-tests were conducted for each indicator within the 3 domains. For knowledge, the proportion of respondents selecting good knowledge was compared between preintervention and postintervention time points, whereas for attitude, the proportion selecting the option agree was compared. For practice, 2-proportion *z*-tests were conducted separately for the 2×/wk and ≥3×/wk frequency categories to distinguish change across levels of practice intensity. Indicators showing statistically significant changes, which also emerged in the qualitative data, were selected for visual presentation in FIGURE 1, FIGURE 2, with significance levels displayed on each panel (∗∗∗*P* < 0.001; ∗∗*P* < 0.01; ∗*P* < 0.05). This approach enabled the identification of the specific nutrition KAP behaviors that showed the greatest response to the intervention in each study area.

## Results

### Overall changes in KAP scores at the block level

**FIGURE 3:**
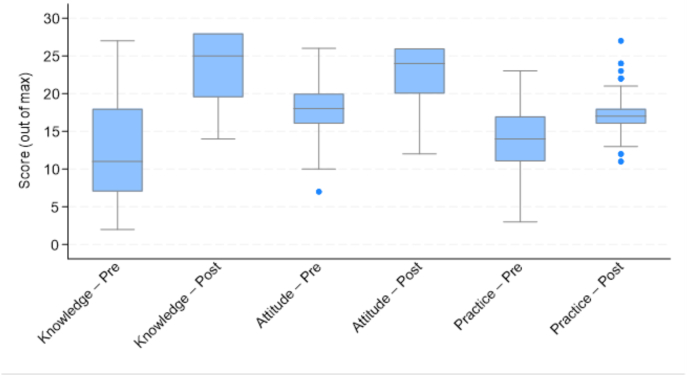
KAP preinterventional compared with postinterventional scores—Chakai. KAP, knowledge, attitudes, and practices.

**FIGURE 4:**
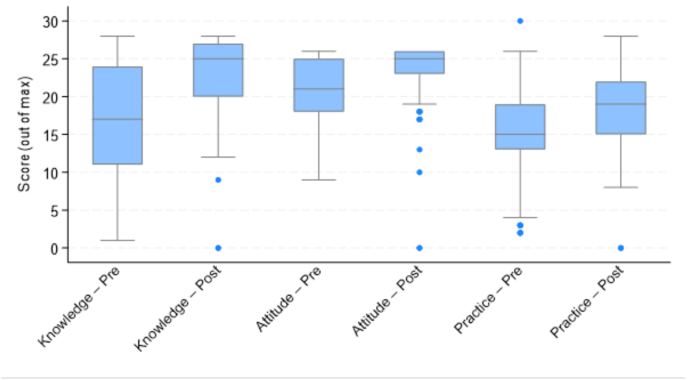
KAP preinterventional compared with postinterventional scores—Shikaripara. KAP, knowledge, attitudes, and practices.

Participants in both Chakai and Shikaripara showed significant improvements in KAP scores after the intervention (*P* < 0.001). Boxplots (Table 1; FIGURE 3, FIGURE 4) illustrate an upward shift in score distributions across all 3 domains (KAP) from preintervention to postintervention. The central line in each box represents the median score, whereas the box indicates the IQR. Postintervention distributions show higher median scores across all domains, with a narrowing of the IQR, suggesting increased participant consensus and a greater consistency in responses. Notably, attitude and practice scores were marginally higher in Shikaripara postintervention, which may reflect the design refinements introduced during phase 2.

**TABLE 1:** Changes in KAP scores before and after intervention in Chakai and Shikaripara.

Block	*n*	K (Pre)	K (Post)	A (Pre)	A (Post)	P (Pre)	P (Post)	K, difference between means (*P*)	A, difference between means (*P*)	P, difference between means (*P*)
Chakai	288	12.73 (6.42)	23.60 (4.40)	17.89 (3.04)	22.53 (3.30)	13.67 (4.49)	17.14 (2.42)	+10.87 (<0.001)	+4.65 (<0.001)	+3.47 (<0.001)
Shikaripara	207	17.27 (6.96)	23.02 (4.92)	20.75 (4.29)	23.74 (3.51)	15.32 (5.22)	18.50 (4.62)	+5.75 (<0.001)	+2.99 (<0.001)	+3.18 (<0.001)

Values are mean (SD).

Abbreviations: K, knowledge (score of 28); A, attitude (score of 26); P, practice (score of 30).

In Chakai, mean knowledge scores rose from 12.73 (SD: 6.42) preintervention to 23.60 (SD: 4.40) postintervention, a gain of +10.87 points (*P* < 0.001), whereas in Shikaripara, mean knowledge scores improved by +5.75 points (*P* < 0.001), from 17.26 to 23.02. The higher baseline in Shikaripara could be attributed to closeness to the district headquarters, access to markets, and stronger collective institutions, partly resulting from a more focused NGO engagement with Adivasi populations (60.5% as compared with 17.2% in Chakai). Gains in attitude and practice scores are also statistically significant across both sites and phases.

### Indicator-level changes: KAP

Although the mean scores provide an overall measure of change, in this section, we explore in further detail indicator-level changes, focusing on practices and knowledge, which offer insight into specific shifts and blockages. The changes visible in the preinterventional and postinterventional surveys (Supplemental Tables 4–6) have been reinforced by qualitative narratives, illustrating growing awareness and application of nutrition principles in daily life.

Across both Chakai and Shikaripara, marked improvements are seen in nutrition knowledge, especially the role of iron in preventing anemia among women. In Chakai, the proportion of participants recognizing the importance of dietary diversity rose from 37% to 93%, and in Shikaripara, awareness that eggs are a good protein source rose from 47% to 85%, whereas the recognition of the need to consume ≥5 food groups rose from 43% to 74%.

Attitudes also improved, particularly around women’s nutrition. Agreement with the statement “women need more iron-rich foods” rose from 67% to >90%. Participants demonstrated a stronger understanding of micronutrients, especially iron, recognized beans and lentils as good sources of fiber, and reported greater adherence to food safety practices such as washing vegetables before cutting them.

On the practice side, there is a significant rise in the consumption of green leafy vegetables, with families increasingly collecting and drying leafy vegetables for year-round use, an activity previously limited to a few households. The proportion of participants planning meals >3 times per week rose from 29% to 67% in Chakai, whereas those reporting never paying attention to meal ingredients fell to <1%.

### Observed changes in dietary practices and local food use

The integration of traditional food knowledge into their diets emerged as a particularly successful outcome of the intervention. Based on our FGDs, semi-structured interviews, and follow-up meetings with the nutrition champions, we found that >65% of the women demonstrated strong recall of recipes introduced during the workshops—such as “peethas” (steamed pancake), “barbetti dal” (black-eyed beans; *Vigna unguiculata*), chutneys, and “sukha machli” (dried fish)—and their nutritional benefits: “iron, vitamin, strength, better immunity” (FGD with women in SV2). Several women noted never having eaten “sajna ke phul ka peetha” (moringa leaf pancake; *Moringa oleifera*) or “makkai bhaat” (maize gruel; *Zea mays*), recipes that were a part of their traditional diet.

“Jonra daka hum do saal baad kha rahe hain, par jab hum chote the toh isi ko bahut pasand karte the. Pata nahi kab ye sab khana chooth gaya, ab shuru karenge” [I am having “Jonra daka” (maize porridge) after almost 2 y and I remembered that I used to like it so much, especially while growing up. I don’t know how and when we stopped eating these foods on a regular basis. We will start now.] Participant in Chakai village (CV)1.

Survey findings further revealed a rise in consumption of green leafy vegetables from 56% to 79% in Shikaripara and from 40% to 60% in Chakai (Figure 1, P8). Women now articulate a clearer distinction between different types of “saag” (greens)—“jungle” (forest), “pahad” (mountain), and “bari” (homestead)—and identified as many as 18 varieties. They shared examples of monsoon greens such as “baibinda saag” (from the forest) and “khantra saag” (from the fields), which they now consciously collect, dry, and preserve, some in powdered form for the nonmonsoon months. Women also reported drying tomatoes for use when market prices rise during off-seasons and increasingly recognized that all parts of the “sahjan” (*M*. *oleifera*) tree are edible and nutritious. Forest produce such as tamarind (*Tamarindus indica*) and “ber” (Indian Jujube; *Ziziphus mauritiana*) leaves, once neglected, are now being collected, dried, and stored for later consumption.

Foods previously avoided because of perceived health risks, such as “ghanghra dal” (black-eye bean; *V*. *unguiculata*) or *sahjan* (*M. oleifera*), once feared to cause weakness or low blood pressure, are now reintroduced into daily diets through recipe demonstrations and follow-ups. As one champion recalled, “Chinti ki chutney pehle bahut khaate the, ab bhool gaye the” (Earlier we used to eat red ant chutney a lot, now we have forgotten), highlighting how training and collective memory have revived traditional practices. Similar stories emerged around “mahua laddoos” (*Madhuca longifolia* or flower of the honey tree) and different forms of “peetha,” including “banwar peetha” (pancakes filled with roasted field rats), all made from locally available ingredients. Homestead gardening has also expanded, with families cultivating tomatoes, gourds, brinjal, and various beans to ensure a steady supply of nutritious food.

The postinterventional data indicated a stronger alignment between nutrition and related attitudes and practices. Participants who expressed greater appreciation for dietary diversity were also more likely to report consuming a wider variety of foods. Meal planning improved from 30% to 67% in Chakai and 19% to 36% in Shikaripara (Figure 1, P2), with women reporting regular inclusion of nutritious local foods including “khatta saag” (a green leafy vegetable), “sahjan” (*M. oleifera*), “sukhi machli” (dried fish), and “dal” (lentils; *Lens culinaris*), alongside potatoes, tomatoes, and aubergine, the latter easily available in local markets and largely affordable (FGD with women in SV2) (Figure 1, P1/P2/P3). Women across the 2 sites noted as follows:

“Pehle dal ya sabzi me se koi ek banate the, ab alag alag tarah ka khana khaate hain” (Earlier we would make either 1 lentil or 1 vegetable. Now we try to eat different varieties of food) and “Dal me paani zyada aur dal kam hota tha, ab soch samajh ke tay karte hain ki kya aur kitna pakana hai” (Earlier it was less lentil and more water, now we are conscious of what and how we are planning and cooking.) FGD with women in Shikaripara Village (SV)1 and CV2.

Both men and women now make more intentional food choices, with women increasingly collecting nutritious forest produce when they can. Eating 3 meals a day was reported as an accepted practice. Many households have adopted healthier cooking practices—using less oil, washing vegetables before cooking, and ensuring balanced portions of lentils, rice, and vegetables.

A notable shift was observed in gendered norms of food equity within households. Earlier, women typically ate last, especially on days when meat, fish, or chicken was prepared, often receiving only the “jhol” (gravy). After the intervention, women reported serving their portions separately or eating whenever hungry. One woman noted a shift in family dynamics: “Meri saas bhi poochti hai ki humko maans mila ya nahi” (My mother-in-law also asks if I got meat or not).

Finally, qualitative findings highlight the importance of Panchayat-level leadership in establishing the foundations of an integrated community-based nutrition model. Both the sarpanch (elected head of the village assembly) and women frontline workers acknowledged the importance of dietary diversity and emphasized the promotion of homestead gardens, through the Panchayat’s “Didi-Bari” scheme, not only as a nutritional strategy but also as a pathway to strengthen food security and self-reliance. One sarpanch noted that the Panchayat is actively facilitating access to seeds and saplings from the block level, ensuring that resources are available to the community when needed. He said, “We can organize regular meetings and awareness-building workshops during the ‘Rozgar Divas’ (Employment Day). This will give us a chance to also talk about interlinkages with other Panchayat schemes that overlap with this intervention.”

Shifts in practices as discussed in the last section, and some attitudes, were driven by improvements in women’s food literacy and knowledge, alongside autonomy in food choices. As 1 woman from SV1 shared, “Hum meeting me ye banana seekhe aur agar dada nahi khate hain tab bhi apne liye bana ke khaate hain” (I learned this recipe during the workshop and even if Dada (her husband) doesn’t eat it, I still make it for myself). Preparations of different kinds of “peetha” became especially popular, fostering a sense of pride in reclaiming traditional and locally foraged recipes (Figure 2, K13). The revival of “banwar peetha” is notable: earlier, many women avoided even acknowledging such foods because of the fear of social judgment.

Peer-to-peer exchange emerged as a concrete knowledge outcome. Women from villages in Shikaripara described how neighbors frequently express curiosity about newly learned recipes:

“Agal bagal ke gaon ki didi log sunti hai toh aati hai poochne kya naya khana banane seekh rahe hain humlog aur woh log bhi seekhna chahti hai.” (They come and ask what new we have learned and what are these recipes? They ask to learn these from us. We share with them whenever we make these.) FGDs in SV1 and SV3.

SHG women, in particular, bring these learnings to their regular meetings, where they actively share recipes, preparation methods, and nutrition-related knowledge. One of the respondents from a village recalled the traditional recipes her grandmother used to prepare—dishes made from forest produce, millets, wild greens, and seasonal roots (Figure 2, K14). These foods, once a part of her daily meals, had slowly disappeared from her kitchen and memory. Through the workshops, she learned that these traditional dishes were rich in nutrients and has not only since begun cooking these recipes at home but also inspiring others in her village.

Intergenerational knowledge sharing was also observed. The nutrition champions mobilized older women in the community to share insights on the nutritional value of traditional foods, help bridge generational gaps and influence young mothers, daughters-in-law, and children to adopt traditional, nutritious diets. In Chakai, elders were found teaching young boys how to hunt field rats after the paddy harvest, once a favorite seasonal activity.

The nutrition champions also reported a growing consciousness around food consumption, particularly higher consumption of fruits, vegetables, seasonal greens, eggs, and iodized salt.

“Pehle pet bharna hi kaafi tha,” (Earlier we would eat only to fill our stomach), one champion shared. “Ab soch samajh ke khana banta hai” (Now we think consciously about what to cook) (FGD with nutrition champions).

Women in Shikaripara continued to practice what they had learned even in the absence of regular follow-ups by the PRADAN team and expressed a strong desire for continued engagement and learning (Figure 2, K1 and K13).

### Challenges remain

Despite the overall gains, challenges remain. Knowledge about vitamin B-12 and vitamin A continued to lag behind other nutrition indicators, with >20% of participants still reporting no knowledge after the intervention (Supplemental Table 4). Although discussed in the workshops, these perhaps remained more abstract compared with an understanding of iron and anemia, not only visible in physical symptoms but also discussed regularly by the frontline health workers, especially during pregnancy.

Some attitudinal ambivalence persisted regarding the feasibility of sustaining diverse diets, particularly in Chakai, where 41% expressed doubts, although recognizing the nutritional benefits and intergenerational significance of local, Indigenous foods in maintaining dietary diversity. This was directly linked to the everyday structural constraint participants confronted, including water scarcity, economic precarity, social stigma, and unequal gender norms. In villages where land ownership is scarce, households depend largely on the market to meet food needs. Water scarcity in the summer reduces the possibility of homestead gardening, a challenge highlighted also by a sarpanch, noting that the rocky terrain has led to a sharp decline in the water table, making year-round self-cultivation difficult to sustain. Nutrition security is inseparable from climate resilience. Persistent financial constraints restrict market purchases. As one woman explained,

“saag mil jata hai, sabzi nahi ho pata, paise ka abhav hai.” (It’s possible to get leafy greens, but we do not manage vegetables because of lack of money.) FGD with women in SV1.

Time poverty emerged as another major barrier, particularly for women who shoulder both domestic and agricultural responsibilities. Even when knowledge and ingredients are available, the time and labor required to forage from the forests or prepare diverse, nutritious meals reduces the frequency of their consumption. “Ghanghra dal (black eyed beans) takes a lot of time to cook,” 1 woman noted. For most women, demanding daily routines shrink the time available for both foraging and cooking.

The persistence of social stigma surrounding Indigenous foods also emerged as a significant challenge. Although some households continue to consume foods such as pigs, red ants, and field rats, discussions around such practices remain suppressed because of caste-based prejudices and perceptions of social status. Quotes from the cooking demonstration workshop reflect these perceptions: 1 woman noted as follows:

“Mera bachha log ye khana nahi khana chahta hai school me dost log majak udayega ya achha khana nahi samjhega sochke” (My children do not eat these foods because they feel their nontribal friends may make fun or not consider them to be good food.)

Similarly, a school-going girl remarked as follows:

“Humko tiffin mein bread-samosa ya bread aur aloo chips achha lagta hai” (I prefer bread and fried snacks or potato chips in my tiffin.)

As men migrate to cities and towns, the influence of urban and dominant-caste food norms become more pronounced, gradually erasing Indigenous foods from public conversation even when still consumed privately. This has contributed to protein inadequacy in diets and whereas the consumption of eggs, fish, and meat is rising, for many, they remain unaffordable. Finally, the relatively low uptake of dairy and nut consumption reflects structural constraints rather than awareness gaps, as many Adivasi populations in India are dairy and lactose intolerant, whereas nuts are not affordable to most [40].

## Discussion

The intervention across Chakai and Shikaripara demonstrates how community-based, culturally grounded nutrition programs can generate meaningful changes in dietary practices, food literacy, and local food systems. Both quantitative and qualitative evidence suggest that the combined strategy of nutrition education, recipe-based interventions, and peer-led mentorship has fostered sustainable shifts in KAP among Santal women, not just around consumption practices but also in relation to food and nutrition security more broadly.

### Reclaiming traditional foods for sustainable consumption and production

The revival and acceptance of traditional foods observed in this intervention reflect a broader process of food sovereignty, strengthening cultural memory, climate-sensitive production, and nutritional resilience, alongside recognizing the potential contribution of Indigenous knowledge to sustainable development more broadly [[41], [42], [43]]. Demonstration workshops and follow-up visits by the nutrition champions have played a critical role in building trust and helping women rediscover local knowledge and reclaim Indigenous practices, what Cheney et al. [44] have called ancestral recipes. These findings align with evidence that community-led food interventions are most effective when they center local knowledge systems rather than imposing external dietary frameworks [21,41,45]. The intervention thus sparked not just dietary changes but also a deeper women-led cultural reclamation of food traditions, knowledge, and health, with shifts in social norms and women’s agency seen as critical for improving nutritional outcomes [[46], [47], [48]].

Communities reflected on their experience during COVID-19 lockdowns: villages located near forests fared significantly better in terms of food availability, diversity, and community care, whereas those dependent on markets struggled [49]. This reinforced the argument that forest-based food systems directly not only provide nutrient-rich foods and incomes for food security but also contribute to ecosystem services for food production, biodiversity protection, and climate change mitigation through carbon sequestration, thereby acting as a critical buffer in times of crisis [[50], [51], [52], [53]]. Investments in traditional, Indigenous food knowledge therefore constitute a form of nutritional resilience.

More broadly, these adaptations reflect an evolving food culture that seeks to integrate nutrition, sustainability, and Indigenous knowledge, while critically engaging with the influence of migration and markets, which have contributed to a growing reliance on ultraprocessed foods [6,42]. Such foods are often associated with status and modernity, despite concerns regarding their nutritional quality and adulteration, highlighted recently by the Lancet Commission on Ultra-processed Foods and Human Health [9,54]. Addressing this dynamic is essential for sustainable dietary improvement in similar low-resource, forest-dependent contexts [55].

### Women’s agency as key to food sovereignty and improved nutrition

The shifts observed in gendered food norms and women’s autonomy in food choices point to a meaningful, if gradual, transformation in household food dynamics. Prior literature establishes that women’s agency and empowerment are foundational to achieving nutrition security and improving household dietary quality [15,46,48]. By building confidence, skills, and collective motivation through hands-on workshops conducted in the local language [16,44,45], the intervention appears to have moved women from passive recipients of food to active decision makers in their households. Celebrating local food traditions through food fairs and documentary screenings, such as the Lahanti Collective’s films on tribal foods, further sparked curiosity and enthusiasm, particularly among youth [16].

The emergence of peer learning networks and intergenerational dialolgue represents a potentially durable mechanism for sustaining dietary change beyond the life of the program. Open discussions across generations have enabled collective learning, support, and a move toward culturally grounded food practices to address health concerns [56,57]. Women continued practicing what they had learned even in the absence of interactions with the NGO team, suggesting that embedding nutritional knowledge within the social and cultural practices of the community increases the likelihood of sustained behavior change [31,[58], [59], [60]]. Such approaches prioritize collaborative decision making with the goal of increasing empowerment and self-efficacy [61], while remaining attentive to the social determinants of health and nutrition, including the environmental context, education, socioeconomic status, access to health care, and experiences of systemic stigma (e.g., racism or weight bias), which can influence nutrition behaviors [62]. Food literacy interventions such as this, drawing on multiple domains of behavior change theory, align with global evidence demonstrating that knowledge and practical skills together can drive dietary change in low-resource contexts [[63], [64], [65], [66]].

### The limits of awareness-based interventions

Although the results demonstrate significant KAP gains, they also underscore the limits of awareness-based approaches in the absence of structural enablers. Time poverty, economic precarity, water scarcity, and the gendered burden of food preparation are not resolved by nutrition knowledge alone. Despite improved knowledge and attitudes, the lower uptake of diverse foods reflects these material constraints, highlighting the need to complement nutrition literacy programs with livelihood support, infrastructure investment, and redistributive approaches to unpaid domestic labor, especially in low-income contexts [14,15,36].

The stigma attached to Indigenous food practices, particularly those associated with lower-caste or forest-dependent identities, poses a subtler but significant barrier. As dominant urban and caste norms continue to shape food aspirations in India, especially through male migration, the cultural gains achieved through this intervention risk erosion unless actively reinforced. Although underresearched, nutrition programs operating in similar contexts must grapple with these dynamics explicitly, rather than treating food choice as a purely individual or knowledge-based matter [67].

Methodologically, the participatory and iterative nature of the intervention required intensive engagement and repeated follow-ups over time, potentially limiting its direct transferability to other contexts without sustained institutional support. In the intervention reported in this study, a potential limitation is the lack of a control group, somewhat restricting our ability to attribute observed changes solely to the intervention. Further, the relatively short follow-up period of 4 months captures early behavioral shifts and not necessarily their long-term sustainability.

## Conclusions: implications for policy and scaling

This study examined whether a community-based, culturally grounded nutrition intervention could improve KAPs among Santal women in Eastern India. The findings demonstrate that it can and that the changes extend beyond measurable KAP scores. These outcomes highlight the potential of participatory, recipe-based approaches to act as effective levers for both nutritional improvements and women’s empowerment in marginalized rural and Indigenous communities. Success is more likely not only when interventions are adapted to local food cultures and ecological conditions but also embedded within existing community and governance structures rather than implemented as standalone projects [68].

Quantitatively, participants across both sites showed significant improvements in all 3 domains (*P* < 0.001). Mean knowledge scores rose by +10.87 points in Chakai and +5.75 points in Shikaripara, with similarly significant gains in attitude and practice scores. Meal planning, dietary diversity awareness, and green leafy vegetable consumption all increased substantially, aligning with evidence that goal setting, problem solving, and social support are effective strategies to facilitate food and health behavior change [69]. Qualitatively, women reclaimed Indigenous foods and practices from which they had gradually been distanced, developed greater autonomy in household food decisions, and built peer-learning networks that continued without external support. Intergenerational dialog around food and nutrition was revived, and a growing alignment between KAPs was observed, with women not only knowing more about nutrition but also actively changing what and how they cook. The take-home message is that when nutrition interventions are rooted in local food cultures, led by community members, especially women, and designed to build skills alongside knowledge, they can generate meaningful and potentially durable change.

Structural barriers, including the seasonality of local foods, water scarcity, market access, women’s time poverty, and persistent stigma around Indigenous diets, alongside weak institutional linkages, however, continue to constrain the sustainability of these changes. Strengthening linkages with local institutions such as Panchayats, Self-Help Groups, frontline workers, and other central and state government programs will be crucial to embedding these practices within local governance and food systems, alongside peer learning and community ownership. Early convergence with schemes such as “Didi-Bari,” which integrates local greens into Panchayat-supported crop planning, and the potential to embed nutrition awareness into existing “Rozgar Divas” (Employment Day) meetings, illustrate how community-led initiatives, when coupled with local governance support, can catalyze sustainable and locally rooted food systems. Sustaining such engagement is critical for enabling and reinforcing behavioral change, peer learning, and local ownership over time.

Greater policy convergence and multisectorial coordination are needed to ensure both scalability and sustainability. “Anganwadi” and school-based nutrition programs (midday meals) should systematically embed local foods in institutional diets. Policy-level stakeholders must recognize the value of locally grounded food strategies for nutrition-sensitive programming, while addressing structural constraints that limit their reach.

Ultimately, the findings underline the need to integrate Indigenous food knowledge and women-led peer education into nutrition-sensitive policies and interventions. By aligning such approaches with the SDGs, particularly Zero Hunger (SDG 2), Good Health and Well-being (SDG 3), Gender Equality (SDG 5), and Responsible Consumption and Production (SDG 12), programs similar to this can contribute meaningfully to building resilient, equitable, and culturally appropriate food systems that strengthen the food security and nutrition of Indigenous communities.

## Declaration of generative AI and AI-assisted technologies in the writing process

The authors declare that no generative AI or AI-assisted technologies were used in the writing of this manuscript.

## Author contributions

The authors’ responsibilities were as follows – NR, AB: have contributed equally to the conceptualization of the paper, research design, and collection of both quantitative and qualitative data, especially in phase 1, review, writing, and editing of the paper; MK: cleaned the KAP survey data for both sites and contributed data analysis including visualization; GK, TB: collected qualitative data in Shikaripara block; and all authors: have read and approved the final manuscript.

## Data availability

Data described in the manuscript, code book, and analytic code will be made available upon request pending application and approval.

## Funding

This study was supported by the EPSRC Global Research Translation Award EP/T015411/1UKRI entitled “Meeting the SDGs” and the University of East Anglia’s Pro-Vice Chancellor’s Fund (PVC, 2022-23, ICF222308, and PVC, 2023-24, ICF232405A) for researching impact. The funders had no involvement in the study design, collection, and analysis of data and posed no restrictions regarding publication. The publication of this paper was supported by the Shakopee Mdewakanton Sioux Community, through a gift to the University of Minnesota from its Seeds of Native Health campaign.

## Conflict of interest

NR reports that financial support was provided by University of East Anglia School of Global Development. All other authors report no conflicts of interest.
