# Supplementary material for: Building on Indigenous Knowledge for Strengthening Food Security and Nutrition: Evidence from a Community-based Intervention in Eastern India
Source: Curr Dev Nutr. 2026 Jul 13;10(8):109434. doi: 10.1016/j.cdnut.2026.109434 (PMC13453556; doi:10.1016/j.cdnut.2026.109434)
Supplement: Supplementary file 1 [file mmc1.docx]

**Supplementary material**

**Table 2. Socio-demographic characteristics of participants in Chakai and Shikaripara**

| **Characteristic** | **Chakai (%)** | **Shikaripara (%)** |
| --- | --- | --- |
| **Age group (years)** |  |  |
| 18–24 | 13.7 | 4.43 |
| 25–34 | 35.0 | 23.3 |
| 35–44 | 27.1 | 34.0 |
| ≥45 | 24.2 | 38.4 |
| **Household size** |  |  |
| 1–3 members | 8.1 | 22.8 |
| 4–6 members | 65.7 | 59.1 |
| ≥7 members | 26.3 | 18.1 |
| **Sex** |  |  |
| Female | 96.0 | 100.0 |
| Male | 4.0 |  |
| **Position in household** |  |  |
| Mother (adult woman) | 80.5 | 97.6 |
| Grandmother | 8.7 | 1.8 |
| Daughter | 4.7 | 0.6 |
| Son/father/grandfather | 4.0 | - |
| Daughter-in-law | 2.0 | - |
| **Education level** |  |  |
| Can read and write | 27.2 | 27.2 |
| Can write only | 25.2 | 26.8 |
| Neither/not reported | 47.7 | 46.0 |
| **Nature of employment** |  |  |
| Farmer | 68.8 | 56.7 |
| Agricultural labourer | 7.7 | 5.1 |
| Farmer & agricultural labourer | 9.1 | 38.2 |
| Not employed | 14.4 | - |

**Table 3: Census-based demographic profile of study blocks (Census 2011)**

| **Indicator** | **Chakai Block, Jamui (Bihar)** | **Shikaripara CD Block, Dumka (Jharkhand)** |
| --- | --- | --- |
| **Total population** | 235,188 | 131,464 |
| **Rural population (%)** | 100% | 100% |
| **Male population (%)** | 52.2% (122,790) | 50.0% (65,723) |
| **Female population (%)** | 47.8% (112,398) | 50.0% (65,741) |
| **Children (0–6 years) (%)** | 18.0% (41,904) | 17.0% (22,931) |
| **Sex ratio (females per 1,000 males)** | 915 | 1,000 |
| **Child sex ratio (0–6 years)** | 964 | 968 |
| **Overall literacy rate (%)** | 57.1% | 57.1% |
| **Male literacy rate (%)** | 69.9% | 68.5% |
| **Female literacy rate (%)** | 42.9% | 45.8% |
| **Scheduled Caste (SC) population (%)** | 17.3% | 3.8% |
| **Scheduled Tribe (ST) population (%)** | 17.2% | 60.5% |
| **Total workers** | 107,517 | 57,175 |
| **Main workers** | 57,416 | 26,176 |
| **Marginal workers** | 50,101 | 30,999 |
| **Cultivators (among main workers)** | 22,982 (40%) | 9,735 (37.2%) |
| **Agricultural labourers (among main workers)** | 25,570 (44.5%) | 6,269 (23.9%) |
| **Dominant social profile** | SC–ST mixed agrarian population | Predominantly ST (tribal) population |
| **Dominant languages (regional)** | Hindi, Khortha | Santali, Khortha, Bengali |

**Table 4: Pre- and post-intervention distribution of nutrition knowledge indicators by study area (Chakai and Shikaripara)**

| **Code** | **Knowledge Indicator** | **Study Area** | **Good Knowledge (%) Pre / Post** | **p-value & significance** | **Some Knowledge (%) Pre / Post** | **No Knowledge (%) Pre / Post** |
| --- | --- | --- | --- | --- | --- | --- |
| K1 | Diverse diet supports good health | Chakai | 37.2 / 92.9 | <0.001 (***) | 35.2 / 5.4 | 27.7 / 1.7 |
|  |  | Shikaripara | 40.1 / 68.2 | <0.001 (***) | 34.8 / 28.7 | 24.9 / 2.9 |
| K2 | Intake of food at least 3 times/day provides sustainable energy | Chakai | 41.3 / 99.3 | <0.001 (***) | 35.9 / 0.0 | 23.5 / 0.7 |
|  |  | Shikaripara | 53.0 / 77.0 | <0.001 (***) | 37.8 / 22.3 | 8.9 / 0.7 |
| K3 | Eating at least five food groups is important for good health | Chakai | 37.2 / 97.3 | <0.001 (***) | 26.6 / 2.0 | 37.2 / 0.7 |
|  |  | Shikaripara | 42.5 / 74.3 | <0.001 (***) | 38.5 / 23.5 | 18.9 / 2.2 |
| K4 | Vitamin B12 is naturally present in foods of animal origin | Chakai | 11.3 / 52.4 | <0.001 (***) | 26.6 / 24.5 | 62.2 / 23.2 |
|  |  | Shikaripara | 13.9 / 40.9 | <0.001 (***) | 51.8 / 33.3 | 34.0 / 25.7 |
| K5 | Vitamins and minerals are important for women of childbearing age | Chakai | 7.2 / 46.6 | <0.001 (***) | 44.7 / 34.9 | 48.5 / 18.5 |
|  |  | Shikaripara | 35.2 / 63.1 | <0.001 (***) | 43.7 / 30.3 | 20.9 / 6.6 |
| K6 | Iron consumption prevents anemia in women | Chakai | 25.9 / 67.1 | <0.001 (***) | 42.3 / 27.2 | 31.7 / 5.7 |
|  |  | Shikaripara | 45.0 / 80.4 | <0.001 (***) | 35.2 / 16.3 | 19.8 / 3.3 |
| K7 | Eggs are a good source of protein | Chakai | 32.4 / 92.3 | <0.001 (***) | 33.5 / 5.0 | 34.1 / 2.7 |
|  |  | Shikaripara | 47.2 / 85.3 | <0.001 (***) | 38.1 / 13.9 | 14.7 / 0.8 |
| K8 | Calcium in milk and dairy products is important for bone health | Chakai | 17.1 / 64.1 | <0.001 (***) | 42.3 / 23.5 | 40.6 / 12.4 |
|  |  | Shikaripara | 36.2 / 66.4 | <0.001 (***) | 32.5 / 24.8 | 31.4 / 8.7 |
| K9 | Use of clean water for cooking prevents diseases | Chakai | 42.3 / 93.3 | <0.001 (***) | 31.4 / 4.7 | 26.3 / 2.0 |
|  |  | Shikaripara | 69.1 / 89.0 | <0.001 (***) | 22.8 / 10.1 | 8.1 / 0.7 |
| K10 | Minerals from meat are good for the immune system | Chakai | 18.1 / 63.8 | <0.001 (***) | 39.9 / 24.2 | 42.0 / 12.1 |
|  |  | Shikaripara | 37.1 / 69.7 | <0.001 (***) | 25.8 / 27.0 | 37.0 / 3.3 |
| K11 | Iodized salt has health benefits | Chakai | 49.8 / 100.0 | <0.001 (***) | 22.5 / 0.0 | 27.7 / 0.0 |
|  |  | Shikaripara | 60.7 / 93.8 | <0.001 (***) | 22.9 / 2.6 | 16.4 / 3.6 |
| K12 | Vitamin A–rich foods improve eye health | Chakai | 32.1 / 83.2 | <0.001 (***) | 32.0 / 11.4 | 35.8 / 5.4 |
|  |  | Shikaripara | 26.3 / 60.7 | <0.001 (***) | 31.3 / 20.0 | 42.4 / 19.3 |
| K13 | Lupung Ara, Peetha, and Ohoy Ara provide high energy | Chakai | 18.8 / 69.8 | <0.001 (***) | 46.4 / 22.1 | 34.8 / 8.1 |
|  |  | Shikaripara | 43.1 / 67.2 | <0.001 (***) | 23.5 / 16.4 | 33.5 / 16.4 |
| K14 | Local foods (ool ka achaar, titua ara/hurhuria ara) are good sources of vitamin C | Chakai | 21.8 / 60.4 | <0.001 (***) | 34.5 / 21.1 | 43.7 / 18.5 |
|  |  | Shikaripara | 38.4 / 70.4 | <0.001 (***) | 24.6 / 13.8 | 37.0 / 15.7 |

**Table 5: Pre- and post-intervention distribution of nutrition attitude indicators by study area (Chakai and Shikaripara)**

| **Code** | **Attitude Indicator** | **Study Area** | **Agree (%) Pre / Post** | **p-value & significance** | **Neutral (%) Pre / Post** | **Disagree (%) Pre / Post** |
| --- | --- | --- | --- | --- | --- | --- |
| A1 | Diversity in the diet is important for overall health | Chakai | 68.9 / 96.3 | <0.001 (***) | 25.3 / 2.0 | 5.8 / 1.7 |
|  |  | Shikaripara | 84.15 / 98.55 | <0.001 (***) | 14.79 / 0.72 | 1.06 / 0.72 |
| A2 | Difficult to consume different types of food (sustaining dietary diversity) | Chakai | 44.4 / 58.4 | <0.001 (***) | 28.3 / 1.0 | 27.3 / 40.6 |
|  |  | Shikaripara | 58.25 / 85.14 | <0.001 (***) | 24.21 / 5.07 | 17.54 / 9.78 |
| A3 | Beans and lentils are good sources of fibre | Chakai | 23.9 / 69.1 | <0.001 (***) | 67.9 / 25.5 | 8.2 / 5.4 |
|  |  | Shikaripara | 63.77 / 91.97 | <0.001 (***) | 25.36 / 6.20 | 10.87 / 1.82 |
| A4 | Vitamins in fruits and vegetables support the immune system | Chakai | 46.4 / 80.2 | <0.001 (***) | 41.6 / 13.8 | 12.0 / 6.0 |
|  |  | Shikaripara | 71.38 / 97.09 | <0.001 (***) | 22.61 / 2.91 | 6.01 / 0.00 |
| A5 | Excess salt in the diet is harmful to health | Chakai | 68.9 / 94.6 | <0.001 (***) | 21.8 / 1.0 | 9.2 / 4.4 |
|  |  | Shikaripara | 80.85 / 87.32 | 0.072 (ns) | 11.35 / 5.43 | 7.80 / 7.25 |
| A6 | Women need more iron-rich foods in their diet | Chakai | 66.6 / 97.7 | <0.001 (***) | 27.0 / 0.7 | 6.5 / 1.7 |
|  |  | Shikaripara | 66.32 / 91.64 | <0.001 (***) | 24.56 / 5.82 | 9.12 / 2.55 |
| A7 | Carrots contain vitamin A that supports eye health | Chakai | 51.2 / 89.6 | <0.001 (***) | 40.6 / 7.1 | 8.5 / 3.4 |
|  |  | Shikaripara | 55.09 / 89.09 | <0.001 (***) | 34.04 / 9.82 | 10.88 / 1.09 |
| A8 | Wholegrains provide a slow release of energy | Chakai | 38.9 / 76.2 | <0.001 (***) | 50.5 / 21.1 | 10.6 / 2.7 |
|  |  | Shikaripara | 55.52 / 79.27 | <0.001 (***) | 28.83 / 15.27 | 15.66 / 5.45 |
| A9 | Food intake should match daily activity level | Chakai | 38.9 / 66.1 | <0.001 (***) | 27.7 / 2.7 | 33.5 / 31.2 |
|  |  | Shikaripara | 58.51 / 79.27 | <0.001 (***) | 24.82 / 15.64 | 16.67 / 5.09 |
| A10 | It is better to wash vegetables before cutting | Chakai | 53.9 / 92.6 | <0.001 (***) | 30.0 / 0.7 | 16.0 / 6.7 |
|  |  | Shikaripara | 71.99 / 93.43 | <0.001 (***) | 18.09 / 4.38 | 9.93 / 2.19 |
| A11 | Foods have different storage methods | Chakai | 57.7 / 97.3 | <0.001 (***) | 32.1 / 0.3 | 10.2 / 2.4 |
|  |  | Shikaripara | 63.27 / 89.49 | <0.001 (***) | 29.82 / 7.97 | 6.91 / 2.54 |
| A12 | Regular hydration is essential | Chakai | 68.9 / 99.7 | <0.001 (***) | 25.3 / 0.0 | 6.5 / 0.3 |
|  |  | Shikaripara | 84.59 / 91.64 | <0.001 (***) | 7.89 / 5.09 | 7.53 / 3.27 |
| A13 | Some foods should not be eaten during pregnancy | Chakai | 36.5 / 68.8 | <0.05 (*) | 43.0 / 10.4 | 20.5 / 20.8 |
|  |  | Shikaripara | 42.70 / 72.26 | <0.001 (***) | 37.72 / 21.53 | 19.57 / 6.20 |

**Table 6: Pre- and post-intervention distribution of nutrition practice indicators by study area (Chakai and Shikaripara)**

| **Code** | **Practice Indicator** | **Study Area** | **Never (%) Pre / Post** | **1×/week (%) Pre / Post** | **2×/week (%) Pre / Post** | **p-value & Significance** | **≥3×/week (%) Pre / Post** | **p-value & Significance** |
| --- | --- | --- | --- | --- | --- | --- | --- | --- |
| P1 | Pay attention to meal ingredients | Chakai | 21.5 / 0.7 | 45.1 / 35.2 | 28.3 / 53.4 | <0.001 (***) | 5.1 / 10.7 | <0.05(*) |
|  |  | Shikaripara | 14.86 / 3.99 | 28.62 / 31.16 | 30.43 / 33.70 | 0.476 (ns) | 26.09 / 31.16 | 0.254(ns) |
| P2 | Plan meals ahead of time | Chakai | 22.5 / 0.0 | 35.2 / 8.4 | 13.0 / 24.5 | <0.001 (***) | 29.4 / 67.1 | <0.001(***) |
|  |  | Shikaripara | 21.15 / 3.62 | 26.52 / 32.61 | 33.33 / 28.26 | 0.264 (ns) | 19.00 / 35.51 | <0.001(***) |
| P3 | Eat eggs/meat/fish | Chakai | 23.2 / 1.7 | 45.4 / 48.0 | 22.2 / 42.3 | <0.001 (***) | 9.2 / 8.1 | 0.639(ns) |
|  |  | Shikaripara | 7.53 / 0.36 | 41.22 / 41.30 | 36.20 / 32.25 | 0.397 (ns) | 15.05 / 26.09 | <0.01(**) |
| P4 | Consume fruits | Chakai | 30.0 / 5.7 | 40.3 / 41.6 | 10.9 / 30.9 | <0.001 (***) | 18.8 / 21.8 | 0.371(ns) |
|  |  | Shikaripara | 37.65 / 16.73 | 33.33 / 26.02 | 20.78 / 21.93 | 0.775 (ns) | 8.24 / 35.32 | <0.001(***) |
| P5 | Eat nuts and seeds | Chakai | 50.9 / 53.4 | 36.9 / 29.5 | 11.3 / 13.4 | 0.444 (ns) | 1.0 / 3.7 | <0.05(*) |
|  |  | Shikaripara | 57.19 / 52.19 | 29.14 / 30.66 | 10.07 / 12.77 | 0.388(ns) | 3.60 / 4.38 | 0.685(ns) |
| P6 | Consume milk and dairy products | Chakai | 49.2 / 48.3 | 35.8 / 32.9 | 12.6 / 14.4 | 0.527(ns) | 2.4 / 4.4 | 0.185(ns) |
|  |  | Shikaripara | 53.60 / 52.36 | 28.06 / 30.55 | 11.51 / 11.27 | 0.939(ns) | 6.83 / 5.82 | 0.673(ns) |
| P7 | Use iodized salt | Chakai | 14.7 / 0.0 | 24.9 / 14.1 | 6.1 / 13.1 | <0.01(**) | 54.3 / 72.8 | <0.001(***) |
|  |  | Shikaripara | 26.35 / 2.93 | 10.11 / 11.36 | 11.19 / 8.79 | 0.416(ns) | 52.35 / 76.92 | <0.001(***) |
| P8 | Eat green leafy vegetables (Vitamin A rich) | Chakai | 17.4 / 0.0 | 29.7 / 17.1 | 39.3 / 60.1 | <0.001(***) | 13.7 / 22.8 | <0.01(**) |
|  |  | Shikaripara | 5.09 / 0.00 | 15.64 / 6.57 | 23.64 / 14.60 | <0.05(*) | 55.64 / 78.83 | <0.001(***) |
| P9 | Feel tired or lack of energy | Chakai | 32.4 / 22.8 | 49.5 / 61.1 | 13.3 / 9.7 | 0.176(ns) | 4.8 / 6.4 | 0.404(ns) |
|  |  | Shikaripara | 25.93 / 11.64 | 27.78 / 30.18 | 18.15 / 24.36 | 0.123(ns) | 28.15 / 33.82 | 0.212(ns) |
| P10 | Access to clean drinking water | Chakai | 0.3 / 3.0 | 1.7 / 13.4 | 4.4 / 8.4 | <0.05(*) | 93.5 / 75.2 | <0.001(***) |
|  |  | Shikaripara | 9.03 / 0.36 | 6.14 / 0.72 | 2.89 / 0.36 | <0.05(*) | 81.95 / 98.55 | <0.001(***) |

**Table 7: Sensitivity Analysis: Comparison of Domain Score Means Before and After Mean Imputation**

| **Domain** | **Timepoint** | **Complete-case n** | **Complete-case Mean (SD)** | **Imputed n** | **Imputed Mean (SD)** | **Difference in Means** |
| --- | --- | --- | --- | --- | --- | --- |
| Knowledge | Pre | 187 | 17.49 (7.06) | 207 | 17.27 (6.96) | 0.22 |
| Knowledge | Post | 196 | 23.52 (4.18) | 207 | 23.02 (4.92) | 0.50 |
| Attitude | Pre | 192 | 20.83 (4.37) | 207 | 20.75 (4.29) | 0.08 |
| Attitude | Post | 199 | 24.03 (2.63) | 207 | 23.74 (3.51) | 0.29 |
| Practice | Pre | 182 | 15.57 (5.20) | 207 | 15.32 (5.22) | 0.25 |
| Practice | Post | 193 | 18.70 (4.28) | 207 | 18.50 (4.62) | 0.20 |

**Note:** *Data shown for Shikaripara (n=207) only. Complete-case means calculated using available data excluding participants with missing values on each variable. Imputed means calculated after item mean imputation for 51 participants with 1–3 missing items. Chakai (n=288) required no imputation as 98.3% of participants had complete data across all 37 items. Maximum possible scores: Knowledge = 28 (14 items × 2); Attitude = 26 (13 items × 2); Practice = 30 (10 items × 3). Differences in means range from 0.08 to 0.50 scale points, representing less than 1.8% of respective scale range*

**Annex - 2**

**Research Instruments**

**FGD with CV and Mentors**

**Key guiding questions:**

- What has been the **follow-up experience** after nutritional awareness and recipe workshops?
- What has been the **key uptake** post workshops and what **changes are visible**?
- What do you think **has facilitated** these changes?
- What are **key challenges** and what is easily getting picked up by women and their families?
- What are the challenges especially in the **availability** of nutritional food?
- What are the **motivations** for people- both who were able to adopt it and those who were not able to?
- What is the **gendered nature of the challenges and motivations** in adapting/not able to adapt the approach?
- How do you think **attitudes and behaviors** are shifting? What have been your observations on the attitude shifts towards accepting these foods?
- Have you observed/noticed if people are able **to exercise/use/make the recipes** taught in the workshops? Are they able to **practice** the training workshops in their everyday cooking? What **is the uptake** **without support**?
- Any **health/nutritional outcomes** observations?
- What are **your learnings** in this process? How often are you able to exercise this in your food intake? How has **your existing knowledge strengthened**?
- What do you think of the project’s **scalability**? And what are **your suggestions for on spread and scale**, especially keeping in mind that is sustainable and practical?

**Panchayat level FGDs**

- What are thoughts, views, on **local food consumption for nutritional enhancement**? A little bit on contextual (Santhal) history, **existing knowledge**, shifts and changes around it?
- What **changes** are responsible for contributing malnutrition in the community?
- What do they think of **this intervention and its approach**- nutritional awareness and local food recipe workshops? Do they feel its needs and **thoughts on its sustainability**? What are your suggestions for scaling?
- What is the status of panchayat/**administrative policy around nutritional awareness** and availability of nutritional foods (instance in mid-day meals, anganbadis, kitchen garden etc.)
- What is the **status of access to funds** to provide for nutritional awareness and foods? Challenges, barriers, supportive/active stakeholders?
- Food availability- supply chain discussion? Local food access and awareness?
- What impact/change can the intervention bring on **health**?
- What support do they seek in incorporating knowledge and attitudinal shifts and bringing in nutritional consumption practices in the everyday lives?

**ASHA, AWW, Sarpanch, Panchayat Sachiv, Poshan sakhi**

- Questions on health outcomes- any **nutritional shifts, changes**?
- **Secondary data** on health shifts/outcomes that is already pre-existing
- How can **the intervention support** health shifts, outcomes?
- What do you think of **this approach**- of local foods and spreading knowledge about the nutritional value of local foods in terms of outcomes?
- What are the health needs that the intervention can support through its approach?

**Anganwadi**

- **Access** of nutritional foods?
- **Bottlenecks in accessing** food availability and providing to the most marginalized
- What is the **supply chain of nutritional food availability** till it reaches the child/mother- what are challenges at each level?
- Can the intervention support in a) **awareness** of creating more access and availability b) providing **regular nutrition knowledge support** for promoting health outcomes?
- Can these foods used during demonstrations be brought to the Anganbari? What is the feasibility of that?

**FGD with the SHG around pre and post survey**

- What have been **the elements of the nutritional awareness** and recipe training process? What have been your **key learnings and behavioral shifts**?
- What **changes have happened in your eating**- food and health practices?
- What are **enablers and barriers**?
- What are the **gender related challenges** that inhibit the practice of such learnings- both for yourself and for other women around you?
- How often and with how many people are you able to **share these learnings with people around you**? Who would be those people? How often are you able to support nutritional practices around you?
- What has been **the processes around knowledge sharing**?
- What are the **most useful things learnt** and what are your suggestions towards this?

**Reflective Questions after conducting the cooking workshop**:

- How did you find the one-day meal plate model that we shared with you today? (If you liked it, why? If you did not like it, why not?)
- Would you adopt this in your own kitchen? (If not, why not?)
- What difficulties do you think you may face in preparing this in your kitchen?
- What did you like the most about what we shared with you today?
- What are your opinions or suggestions to improve this further, so that more and more people can prepare it in their own kitchens?

**KAP Survey Tool**

Please give the following information to the respondent and obtain their consent to participate in the survey:

First of all, I would like to thank you for taking out the time to come here today. My name is _________ and I am from _______ village.

Two years ago, we collected information from people in CV1, CV2, and CV3 panchayats about their food and eating habits. We also sent samples of those food items to a laboratory for testing. Based on this, we have prepared a 9-day seasonal meal plan for you. This plan is made using foods that are already part of your everyday diet and are easily available in the village. Each day’s meal plan is designed to provide complete nutrition to the body.

We will share about these 9 meal plans with you, and after you listen to and understand them, we would like to know your opinions and preferences — whether you like them, whether you would eat them to make your diet more nutritious, and whether you think you may face any difficulties in adopting them.

Before sharing the meal plans, we will conduct a survey with you in which we will try to understand your eating habits, food-related customs and traditions passed down over time, cooking practices, and your thoughts and beliefs regarding food. This survey will take about 20–25 minutes.

There are no right or wrong answers to the questions you give; we simply want you to answer based on your understanding and experience. We want to conduct this survey with you because we believe your understanding of food and eating practices will help us better understand this place and its traditions.

If you do not wish to participate, you may let us know. You may stop us at any time to ask questions, or if you feel uncomfortable answering any question, please tell us. Your responses will be kept confidential and will not be shared with anyone. If we use this information for research purposes, we will remove your name and any information that could identify you.

If you have any questions about what I have just explained, you may ask them now.

Would you like to participate in this survey?

| **ATTITUDE / AWARENESS**  *Share your opinion on the statements below* | | | |
| --- | --- | --- | --- |
|  | Agree | Neutral | Disagree |
| Diversity in the diet is important for overall health |  |  |  |
| Difficult to consume different types of food (sustain diversity in diet) |  |  |  |
| Beans and lentils are good sources of fibre |  |  |  |
| Vitamins in fruits and vegetables support the immune system |  |  |  |
| Excess salt in the diet is harmful to health |  |  |  |
| Women need more iron rich foods in their diet |  |  |  |
| Carrots contain Vitamin A that supports eye health |  |  |  |
| Wholegrains provide a slow release of energy |  |  |  |
| Food intake should match daily activity level |  |  |  |
| It’s better to wash vegetables before cutting |  |  |  |
| Foods have different storage methods |  |  |  |
| Regular hydration is essential |  |  |  |
| Some foods should not be eaten during pregnancy |  |  |  |

| **KNOWLEDGE**  *Please tick the box that most closely matches your level of knowledge* | | | |
| --- | --- | --- | --- |
|  | No knowledge | Some knowledge | Good knowledge |
| Diverse diet supports good health |  |  |  |
| Intake of food at least 3 times/day provides sustainable energy |  |  |  |
| Eating at least 5 food groups is important for good health |  |  |  |
| Vitamin B12 is naturally present in foods of animal origin |  |  |  |
| Vitamins & Minerals are important for women of childbearing age |  |  |  |
| Consumption of iron is important to prevent anaemia in women |  |  |  |
| Eggs are a good source of protein |  |  |  |
| Calcium in milk and dairy is important for bone health |  |  |  |
| Sourcing clean water for cooking protects against diseases |  |  |  |
| Minerals in meat is good for the immune system |  |  |  |
| Iodized salt has health benefits |  |  |  |
| Vitamin A rich foods improves eye health |  |  |  |
| Lupung Ara Peetha/Ohoy Ara provides you high Energy |  |  |  |
| Local foods like “ool ka achaar” and “titua ara/hurhuria ara” are good sources of Vitamin C |  |  |  |

| **PRACTICES**  *Share your habitual intake (frequency of consumption) of the different food items* | | | | |
| --- | --- | --- | --- | --- |
|  | Never | 1x/week | 2x/week | >3x/week |
| How often do you pay attention to your meal ingredients |  |  |  |  |
| How often do you plan your meal ahead of time |  |  |  |  |
| How often do you eat eggs / meat / fish |  |  |  |  |
| How often do you have fruit |  |  |  |  |
| How often do you eat nuts and seeds |  |  |  |  |
| How often do you have milk / dairy |  |  |  |  |
| How often do you use iodized salt |  |  |  |  |
| How often do you eat green leafy vegetables rich in Vit A |  |  |  |  |
| How often do you feel tired / out of energy |  |  |  |  |
| How often do you get access to clean drinking water |  |  |  |  |

| Demographic data | | | | | | | |
| --- | --- | --- | --- | --- | --- | --- | --- |
| Name of Participant | |  | | | | | |
| Address | |  | | | | | |
| Age (years) |  | Date Of Birth (DD/MM/YYYY) | | | |  | |
| Gender | | Male | |  | Female | |  |
| Status in family | | Grandmother/mother | | Grandfather/father | Daughter / son | |  |
| Education level | | Read | | Write | Both | | |
| Please indicate if this form was completed before or after the intervention | | | | | | | |
| Pre-intervention | | | Post-intervention | | | | |
| Date: | | Name of the Interviewer | | | | | |
